# Reverse engineering of Ayurvedic lipid based formulation, ghrita by combined column chromatography, normal and reverse phase HPTLC analysis

**DOI:** 10.1186/s12906-015-0568-9

**Published:** 2015-03-13

**Authors:** Selvakumar Duraipandi, Vijaya Selvakumar, Ng Yun Er

**Affiliations:** Department of Pharmaceutical Chemistry, School of Pharmacy, Taylor’s University Lakeside, 47500 Subang Jaya, Malaysia

**Keywords:** Guggulu tiktaka Ghrita, HPTLC, Ayurveda, Chromatography

## Abstract

**Background:**

Ghritas are ayurvedic lipid based preparations in which oil or ghee is boiled with prescribed kasaya (polyherbal decoction) and kalka (fine paste of herbs) until the evaporation of aqueous phase transfers the contents into oily phase. The polyherbal decoction used in the preparation predominantly contains water soluble Active Botanical Ingredients (ABIs).

**Methods:**

The column chromatography was used to fractionate the ghrita into polar and non-polar fractions on silica gel as adsorbent using petroleum ether and mixture of ethanol, methanol & water as eluents. These fractions were further analysed by normal and reverse phase HPTLC analysis for the presence of the contents and its polarity.

**Results:**

The results showed that all the ABIs present in the formulation were polar since the fractionated non-polar fraction did not show the presence of any active botanical ingredients on normal and reverse phase HPTLC analysis.

**Conclusions:**

The ayurvedic system of medicine has got its own technique of incorporating the polar contents into a lipid base for enhanced absorption and delivery of the ABIs at targets

## Background

Ayurveda is a holistic system of medicine that originated from the Indian subcontinent 3000 years ago [1,2]. The main texts on ayurvedic medicine are Caraka Samhita, Sushruta Samhita, and Astanga Hridaya which describes the treatment and ayurvedic medicaments [1]. The term ayurveda means “Science of Life” and some of the very early written documentations on the practice of ayurveda can be traced back to the verses in Rig Veda (1500 BC) [2]. The ayurvedic pharmaceutics comprises of five basic types of formulations namely “Pancavidha Kasaya” from which the different dosage forms were developed [3]. Ghrita, one of its dosage forms utilized cow ghee; to transfer the contents of polyherbal aqueous extract (polyherbal decoction, kasaya) into the lipid base thereby enhancing the absorption and delivery of the contents and also to extend the shelf life [3,4].

The lipid based formulations in the traditional system of medicine, ayurveda, utilizes ghee (butter oil), and taila (edible oils) for the preparation of different dosage forms falling under “Sneha Kalpana” according to ayurveda [5]. Ghritas were preparations in which oil or ghee was boiled with prescribed kasaya and kalka (fine paste of herbs) according to the formula until the aqueous portion evaporated and this process is called “avartana”. This process ensured transfer of the active botanical ingredients (ABIs) into the oil base [3,6]. Some researchers claimed that these preparations were drug delivery systems to deliver poorly water soluble contents or in other words, the contents that have good lipid solubility [4,6-10]. On the contrary, the ABIs transferred from the decoctions should be polar, hydrophilic components since the extraction was made by boiling the herbals in water [3].

In such a case it was assumed that the lipid base contained these polar constituents (hydrophilic) of kasaya in a form other than that of a solubilized form. Since there was no known surfactants added and also the water was completely evaporated, the final formulation did not show any separations as solids or liquid, instead appeared as monophasic liquid, it was thought that the hydrophilic contents may be entrapped in a micro/nano vesicular form which may not appear to the naked eye. The ayurvedic system has got its own technique of incorporating the polar contents into nonpolar medium to control the delivery of the polar active ingredients effectively at targets. Thus, to prove the conceived hypothesis it was designed to analyse and demonstrate that the contents of these lipid based formulations are polar in nature. Hence, we chose to work with a model ghrita, Guggulu tiktaka ghrita (GTG) as a representative of this group of formulation in ayurvedic formulary to prove the postulation that the polarity of the major ABIs are hydrophilic in nature.

Guggulu tiktaka ghrita is widely used as medicine and also in preparatory procedure called snehakarma for the treatment of deep seated ulcers and abscess, sinus, asthma, rhinitis, cough and cold, cardiac diseases and gout [8,9].

The decoction of the major contents *Azardica indica, Trichosanthes dioica, Solanum xanthocarpum, Tinospora cordifolia, Adhatoda vasica* were prepared by boiling in water. The ground paste prepared from the following botanicals in water *Cyclea peltata/Cissampelos pariera, Embelia ribes, Cedrus deodara, Piper chaba, Hordeum vulgare, Zingiber officinalis, Curcuma longa, Anethum sowa, Piper chaba, Saussurea lappa, Zanthoxylum alatum, Black pepper, Holarrhena antidysenterica, Trachyspermum ammi, Plumbago zeylanica, Picrorrhiza kurroa, Purified Semecarpus, anacardium, Acorus calamus, Piper longum – root, Pluchea lanceolata, Rubia cordifolia, Aconitum heterophyllum, Aconitum species, Yavani – Trachyspermum ammi* and sodium bicarbonate. The purified *Commiphora mukul (guggulu),* and Go-ghrita (cow ghee) which makes the lipid base were mixed with the prepared decoction and the fine paste of botanicals and then boiled until the evaporation of water to form the final formulation [8,9].

## Methods

### Materials

All the reagents and solvents used were analytical grade which were purchased from Sigma-Aldrich. The model ghrita “Guggulu tiktaka ghrita (GTG)” was purchased from AVN Arogya Ayurvedic Pharmacy, India. The silica gel (60-120 mesh) for chromatography was purchased from Merck, India. The glass column fabricated with sintered glass filter was supplied by Borosil, India. Pre-coated silica gel GF_254_ aluminium plates for normal phase and the pre-coated RP-HPTLC silica gel 60 RP-18 F_254_s aluminium plates for reverse phase chromatography were purchased from Merck, India to carry out HPTLC analysis on CAMAG HPTLC system, Switzerland.

### Column Chromatographic fractionation of ghrita (Medicated Ghee)

For the separation of ABIs, the column chromatography was employed in which 30 cm long and 10 mm internal diameter glass column was used and the column was rinsed with acetone, dried and packed with the silica gel for column chromatography in petroleum ether [11-13]. Chromatographic separation of the ghrita into fractions was performed by elution with petroleum ether (40-60) under gravity until it did not show the elution of fat content of the ghee, which formed the base for preparing the ghrita monitored by TLC. The separation was then followed by elution with ethanol: methanol (6:4) and then by methanol: water (4:1) until the fractions did not show the presence of ABIs contents in TLC using toluene: ethyl acetate: methanol (7:2:1) as a mobile phase observed under UV light at 254 and 366 nm.

The fractions thus collected were designated as ‘Fraction A’ for the petroleum ether fraction and ‘Fraction B’ for the combined fraction of ethanol: methanol and methanol: water fractions. The TLC was performed to confirm the presence or absence of ABIs of the ghrita in the fractions with reference to the ghrita itself.

The optimized quantities of the adsorbents and solvents required to perform the column chromatographic separation of GTG were given below.

Column: Silica gel (60-120 Mesh), 150 g; Solvents: Petroleum Ether: 400 mL; Ethanol: Methanol (6:4): 200 mL; Methanol: Water (4:1): 100 mL.

### HPTLC Analysis

#### Normal Phase

2 μL samples were applied as 8 mm bands in four tracks (Track 1 - Ghee; Track 2 - Ghrita; Track 3 - Fraction A; Track 4 - Fraction B) on pre-coated silica gel 60 GF_254_ aluminium plates (10 × 10 cm) with the help of Linomat 5 applicator attached to CAMAG HPTLC system, which was programmed through WINCATS software. The detection was performed using Densitometry TLC Scanner 3 at 254 and 366 nm in UV cabinet. The plates were developed in the TLC chambers pre-saturated with mobile phase [Toluene: Ethyl acetate: Methanol (7:2:1)].

#### Reverse Phase

2 μL samples were applied as 8 mm bands in four tracks (Track 1 - Ghee; Track 2 - Ghrita; Track 3 - Fraction A; Track 4 - Fraction B) on pre-coated silica gel 60 RP-18 F_254_s aluminium plates (10 × 10 cm) with the help of Linomat 5 applicator attached to CAMAG HPTLC system, which was programmed through WINCATS software. The detection was performed using Densitometry TLC Scanner 3 at 254 and 366 nm in UV cabinet. The plates were developed in the TLC chambers pre-saturated with mobile phase [Methanol: Water: Glacial Acetic acid (8:2:0.1)].

#### Visualization

The HPTLC analysis was carried out using the above conditions and the developed plates were visualized under UV light and densitometric scanning was performed to obtain the R_f_ values and corresponding concentration of the spots (AU) [14].

## Results and discussion

Since these preparations utilized decoctions of polyherbal mixtures which may contain predominantly polar components, one should wonder how these polar ABIs, mostly soluble in polar solvents like water, methanol were incorporated into a non-polar lipid medium without specialized adjuncts (surfactants) added to them. Upon processing they mixed to form a monophasic oily liquid without any distinct layers even though it contained ABIs of the kasaya. Since hydrophilic (polar) substances used to have poor lipid solubility, they would have been not solubilized in the oily phase but on the contrary, the finished formulation did not show any separations instead formed a monophasic oily liquid.

From the results it was observed that GTG, a model ghrita formulation taken for this study, can be fractionated into polar and nonpolar fractions by using column chromatography on silica gel eluted with respective polar and non-polar solvents. The optimized solvents which can fractionate the ghrita into nonpolar and polar fractions were petroleum ether (40-60) and a combination of ethanol, methanol & water respectively. The extractive values of the fractions A and B were calculated based on the weight obtained after evaporating the solvent under reduced pressure, was presented in Table 1.

**Table 1:** **Extractive values of the solvents obtained after fractionation**

**Ghrita**		**Fraction A (Nonpolar)**			**Fraction B (Polar)**		
**Volume (mL)**	**Weight(g)**	**Volume (mL)**	**Weight (g)**	**Percentage (%)**	**Volume (mL)**	**Weight (g)**	**Percentage (%)**
75	65	56.7	49.14	75.60	17	14.62	22.49

1 mL of Ghrita is equivalent to 0.8605 g of the Ghrita.

**Figure 1:**
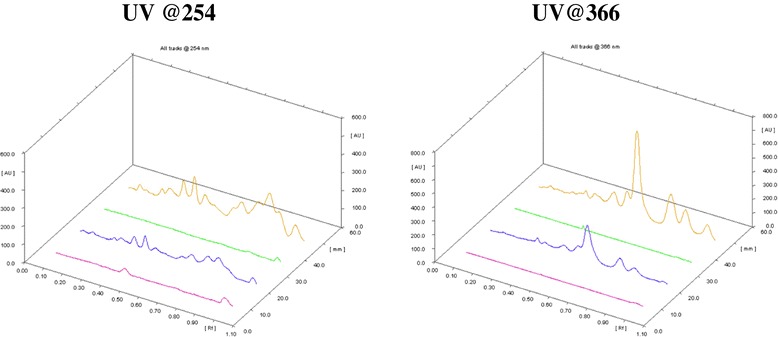
**Normal phase HPTLC chromatograms.** 3D overlay chromatograms showing the behaviour of Ghee (Track 1), Ghrita (Track2), Fraction A (Track 3) and Fraction B (Track 4) viewed under UV light @254 and @366 nm respectively.

**Figure 2:**
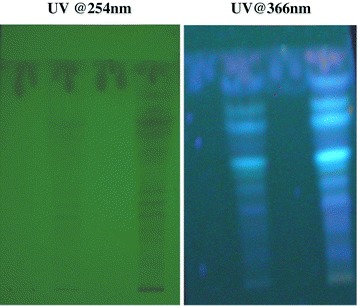
**Visualization of HPTLC plates under UV light @254 nm and @366 nm.** The visualization of the developed silica gel GF_254_ aluminium plates observed under UV light @254 and @366 nm showing Ghee (Track 1), Ghrita (Track2), Fraction A (Track 3) and Fraction B (Track 4) respectively.

**Figure 3:**
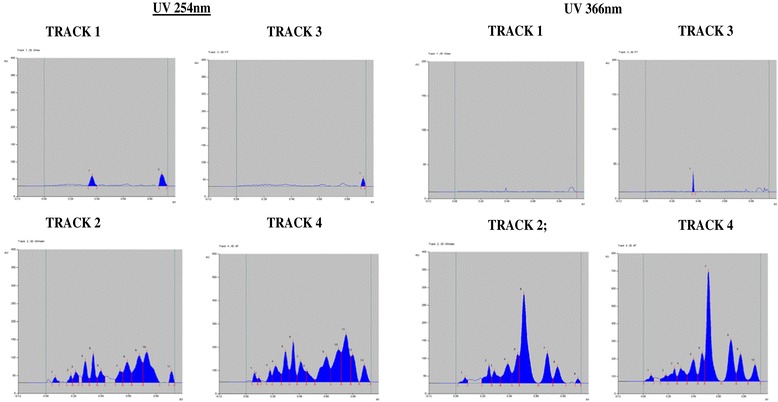
**Normal phase HPTLC densitograms.** Densitograms showing the presence and absence of ABIs in Ghee (Track 1), Ghrita (Track2), Fraction A (Track 3) and Fraction B (Track 4) observed under UV light @254 and @366 nm respectively.

Since ghrita was prepared from mixing ghee, polyherbal decoction and paste of herbs and was expected to contain lipid soluble medicinal contents. However, in the column chromatographic fractionation and normal phase HPTLC analysis on the fractions obtained from the column showed that fraction A, the non-polar fraction did not show the presence of ABIs corresponding to lipid soluble medicinal content from polyherbal extracts. But, in the HPTLC analysis of fraction B, the polar fraction showed presence of ABIs and produced a similar chromatographic pattern as that of the original ghrita in the given solvent system. The HPTLC chromatograms of Track 1 and Track 3 have clearly showed the absence of the ABIs which corresponded to ghee and fraction A respectively whereas the chromatograms of Track 2 and Track 4 marked the presence of ABIs which corresponded to the ghrita formulation and the fraction B respectively. The HPTLC overlay chromatograms of ghee, ghrita and fractions A and B were shown in Figure 1. The visualization under UV light at 254 and 366 nm were shown in Figure 2. The densitograms of the spots developed on the HPTLC plates were shown in Figure 3. From the results it was observed that the ABIs were completely eluted into the polar fraction and it was proved from the normal phase HPTLC analysis of fractions along with ghee and GTG that they were polar ingredients.

**Figure 4:**
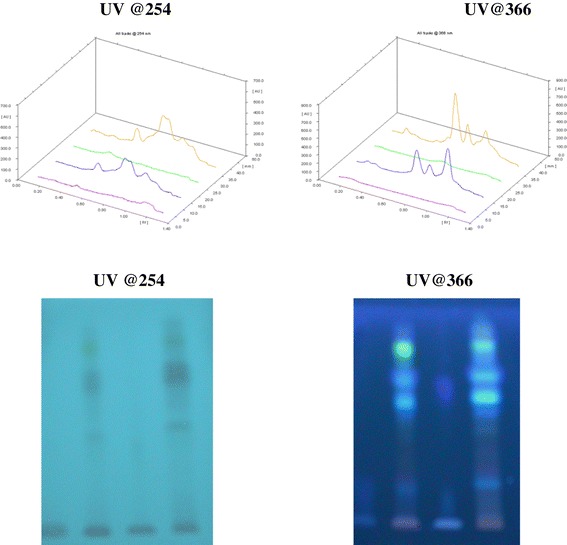
**Reverse phase HPTLC chromatograms and visualization.** 3D overlay chromatograms of Ghee (Track 1), Ghrita (Track2), Fraction A (Track 3) and Fraction B (Track 4) and visualization of the developed silica gel 60 RP-18 F_254_s aluminium plates observed under UV light @254 and @366 nm showing Ghee (Track 1), Ghrita (Track2), Fraction A (Track 3) and Fraction B (Track 4) respectively.

**Figure 5:**
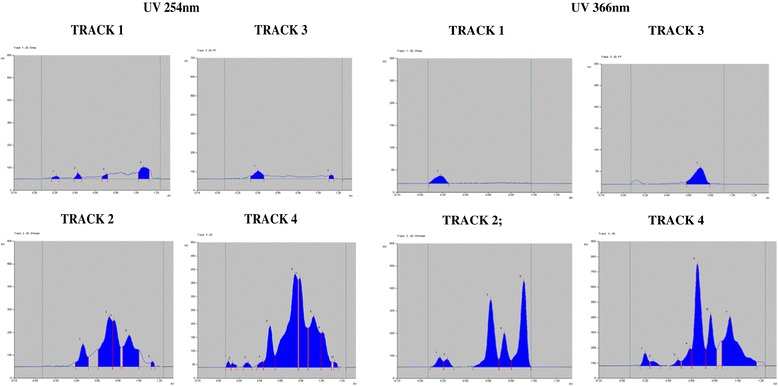
**Reverse phase HPTLC densitograms.** Densitograms showing the presence and absence of ABIs in Ghee (Track 1), Ghrita (Track2), Fraction A (Track 3) and Fraction B (Track 4) observed under UV light @254 and 366 nm respectively.

To further substantiate the claim, the RP-HPTLC analysis was performed on RP-HPTLC silica gel 60 RP-18 pre-coated plates using methanol: water: glacial acetic acid (8:2:0.1) as the mobile phase. As expected fraction A showed the absence of ABIs whereas fraction B showed the full spectrum of the ABIs and produced similar chromatographic pattern as that of GTG under same chromatographic conditions. The HPTLC overlay chromatograms of ghee, ghrita and fractions along with the visualization under UV light at 254 and 366 nm were shown in Figure 4. The densitograms of the spots developed on HPTLC plates were shown in Figure 5.

The normal phase HPTLC has shown similar chromatographic spectrum in fraction A (Track 3) and Ghee (Track 1) when visualized after spraying various destructive spray reagents and found no spot corresponded to the spots that appeared in ghrita and fraction B. The reverse phase HPTLC also has shown similar results which again suggests that the ABIs of the GTG were polar and they were thought to be dispersed in the lipid base in an unknown technique by Ayurveda.

The extractive values of ghrita in two different fractions obtained from column chromatography showed that fraction A was accounted for major portion (75.60%) of the ghrita which did not show the presence of any active botanical ingredient whereas fraction B was accounted for minor portion (22.49%) which showed the presence of all ABIs in the given chromatographic window which includes the major biomarkers Guggulsterones E and Z contained in the selected formulation. The results were presented in Table 1.

## Conclusions

The study proved that the guggulu tiktaka ghrita (GTG), the model ghrita taken for the analysis was containing predominantly polar ABIs incorporated into the ghee which forms the lipid base. The ayurvedic system of medicine has got its own technique of incorporating the polar, hydrophilic contents into a lipid base for enhanced absorption and delivery of the ABIs at targets. Further research on the architecture of this group of formulations will provide more information on the type of delivery system.
